# The effect of dapagliflozin on uric acid excretion and serum uric acid level in advanced CKD

**DOI:** 10.1038/s41598-023-32072-y

**Published:** 2023-03-24

**Authors:** Yukimasa Iwata, Shoki Notsu, Yushi Kawamura, Waka Mitani, Shinjiro Tamai, Madoka Morimoto, Masafumi Yamato

**Affiliations:** Department of Nephrology, Sakai City Medical Center, Osaka, Japan

**Keywords:** Chronic kidney disease, Endocrinology

## Abstract

Sodium–glucose cotransporter 2 inhibitors (SGLT2i) exhibit renoprotective effect in patients with chronic kidney disease (CKD) and reduce serum uric acid (UA) in patients with diabetes mellitus. However, it is not clarified whether SGLT2i reduce serum UA levels in patients with advanced CKD. This study aimed to investigate the impact of SGLT2i on change in serum UA levels in patients with advanced CKD. Data of 121 Japanese patients with CKD who were newly administered 10 mg dapagliflozin in our department between August 2021 and August 2022 were analyzed. Changes in UA and fractional excretion of UA (FEUA) were analyzed using multiple regression analysis. Of 75 patients, 21 (28.0%) patients, 24 (32.0%) patients, 29 (38.7%) patients, and 1 (1.3%) patient were categorized as having CKD stage 3a, 3b, 4, and 5, respectively. The median age was 67 years, and 72.0% were male. 23 (30.7%) of patients had diabetes mellitus. The median estimated glomerular filtration rate, serum UA, and FEUA were 35.7 mL/min/1.73 m^2^, 6.4 mg/dL, and 6.76%, respectively, at the time of dapagliflozin administration. After administration, serum UA decreased to 5.6 mg/dL and FEUA increased to 9.22%. Dapagliflozin increases FEUA and reduces serum UA levels in patients with advanced CKD.

## Introduction

Chronic kidney disease (CKD) is a global concern, affecting more than 10 million patients in Japan and 700 million worldwide^1,2^. Renin angiotensin aldosterone system inhibitors have been shown to reduce urinary protein levels and delay CKD progression^3,4^. Recently, it has been shown that sodium–glucose cotransporter 2 inhibitors (SGLT2i) have a renoprotective effect^5–7^. Although the mechanism of their renoprotective effect is believed to involve the reduction of intra-glomerular pressure by tubular glomerular feedback^8^, SGLT2i have pleiotropic effects and their mechanism of renal protection has not been fully revealed^9^.

Serum uric acid (UA) and renal outcomes are not fully understood; however, it has been reported that allopurinol reduces serum UA levels and improves renal outcomes^10,11^. On the other hand, allopurinol did not improve renal outcomes^12,13^. One of the pleiotropic effects of SGLT2i is reduction of serum UA in patients with type 2 diabetes mellitus (DM) whose estimated glomerular filtration rate (eGFR) was maintained at > 60 ml/min/1.73 m^2^^14–17^; however, the effect of lowering serum UA levels by SGLT2i in patients with CKD whose renal function reduced to ˂60 ml/min/1.73 m^2^ is unclear.

Dapagliflozin 10 mg is the only drug approved for the treatment of CKD with or without DM in Japan as SGLT2i and we analyzed patients who were administered dapagliflozin 10 mg.

Approximately 70% of serum UA is excreted into the urine and the rest is thought to be excreted into the intestinal tract. The renal transport system such as urate transporter 1 (URAT1), adenosine triphosphate-binding cassette transporter G2 (ABCG2), glucose transporter 9 (GLUT9) isoform2 plays an important role in the regulation of serum UA level. SGLT2i increase expression of GLUT9 and ABCG2^18,19^.

Thus, this study aimed to investigate the effect of SGLT2i in lowering serum UA levels in patients with CKD. In addition, this study aimed to examine the effect of SGLT2i in the change in urinary UA excretion.

## Methods

### Patients

This study was conducted in accordance with the Declaration of Helsinki guidelines and approved by institutional review board of Sakai City Medical Center (No. 21-261). All patients were provided with the option to opt out of the study. The need for informed consent was waived using our hospital’s opt-out method.

This was a single-center retrospective cohort study. A total of 121 Japanese patients with CKD who were newly administered dapagliflozin 10 mg in our department from August 2021 to August 2022 and were followed-up for at least 2 weeks were enrolled in the study. Thirty-four patients were excluded from the analysis owing to changes from other SGLT2i (n = 3) or dapagliflozin dose (n = 2). In addition, patients with changes in the dose of diuretics (n = 3) and antihyperuricemic agents (n = 3), and those whose serum UA level was not measured before or after dapagliflozin 10 mg administration (n = 17) were also excluded. Twelve patients with CKD stages 1 and 2 were excluded from the study.

**Figure 1:**
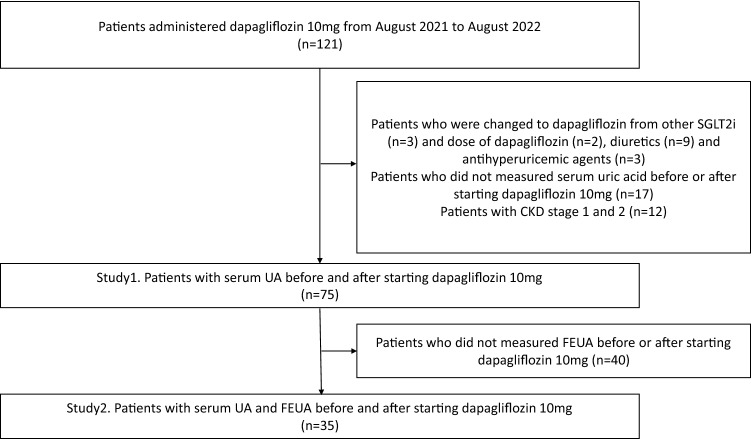
The flow chart of the study population.

In study 1, 75 patients were retrospectively studied (Fig. 1).

Furthermore, patients whose fractional excretion of uric acid (FEUA) was not measured before or after dapagliflozin 10 mg administration (n = 40) were excluded from the study. A total of 35 patients were included in Study 2.

### Data collection and definition

We collected the data of 121 patients from the electronic medical charts of Sakai City Medical Center, including demographics (sex and age), medications, comorbidities (history of heart failure and DM), and clinical and laboratory variables (blood pressure, pulse rate, serum creatinine, blood urea nitrogen, UA, hemoglobin, albumin, FEUA, and proteinuria). FEUA was calculated as ($100 \times urine\; UA \times serum\;creatinine \div serum\;UA \div urine\;creatinine$). eGFR was estimated using the formula for Japanese people $\left( {eGFR \left[ {{\text{mL}}/{\text{min}}/1.73\;{\text{m}}^{2} } \right] = 194 \times serum creatinine - 1.094 \times age - 0.287 \times 0.739 \left[ {for females} \right]} \right)$^20^. Patients were divided into four groups according to their renal function and CKD category (G3–G5).

### Outcomes

This study’s primary and secondary outcomes were, respectively, changes in serum UA and FEUA after administration of dapagliflozin 10 mg.

### Statistical analyses

Continuous variables are presented as medians (interquartile range [IQR]) and were compared using the Kruskal–Wallis test. Categorical variables are presented as numbers and percentages and were compared using the Fisher’s exact test. Changes in UA and FEUA were compared using the Wilcoxon signed-rank test and Kruskal–Wallis test.

Multiple regression analysis was used to estimate the independent factors associated with changes in UA and FEUA.

Spearman’s rank correlation coefficient was used to evaluate an association between change in FEUA and serum UA.

All statistical analyses were performed using EZR software (Saitama Medical Center, Jichi Medical University, Saitama, Japan)^21^.

## Results

### Baseline characteristics

At administration of dapagliflozin 10 mg, 21 (28.0%) patients, 24 (32.0%) patients, 29 (38.7%) patients, and 1 (1.3%) patient were categorized as having CKD stages 3a, 3b, 4, and 5, respectively (Table 1). Patient characteristics and baseline laboratory data for each group are shown in Table 1.

**Table1:** Patient characteristics and laboratory data at starting dapagliflozin 10 mg by CKD stage.

	All patients n = 75	CKD stage 3a n = 21	CKD stage 3b n = 24	CKD stage 4 n = 29	CKD stage 5 n = 1	*p* value
Male (%)	54 (72.0)	15 (71.4)	21 (87.5)	17 (58.6)	1 (100.0)	0.059
Age	67 [57–77]	62 [53–67]	71 [60–77]	73 [62–79]	38 [38–38]	0.041
Comorbid condition						
Heart failure (%)	3 (4.0)	0 (0.0)	0 (0.0)	3 (10.3)	0 (0.0)	0.175
Diabetic mellitus (%)	23 (30.7)	6 (28.6)	8 (33.3)	9 (31.0)	0 (0.0)	0.904
Clinical and laboratory variables						
Systolic blood pressure (mmHg)	133 [122–140]	130 [118–138]	137 [125–145]	135 [122–140]	116 [116–116]	0.184
Diastolic blood pressure (mmHg)	75 [68–83]	82 [72–85]	76 [68–81]	71 [66–77]	74 [74–74]	0.254
Pulse rate (bpm)	79 [70–84]	82 [73–86]	75 [69–87]	78 [69–89]	76 [67–88]	0. 856
Creatinine (mg/dL)	1.49 [1.23–1.95]	1.12 [1.01–1.23]	1.47 [1.33–1.58]	2.01 [1.91–2.24]	4.11 [4.11–4.11]	< 0.001
Estimated glomerular filtration rate (ml/min/1.73m^2^)	35.7 [26.2–46.3]	51.3 [47.6–55.0]	38.4 [34.8–42.4]	24.7 [20.67–27.2]	14.6 [14.6–14.6]	< 0.001
Blood urea nitrogen (mg/dL)	24 [18–32]	16 [16–18]	23 [20–27]	33 [27–41]	43 [43–43]	< 0.001
Uric acid (mg/dL)	6.4 [5.6–7.0]	6.1 [5.5–7.2]	6.5 [5.9–6.8]	6.5 [5.8–7.0]	6.8 [6.8–6.8]	0.837
Hemoglobin (g/dL)	12.6 [11.1–13.9]	13.7 [12.8–14.4]	12. 5 [10.9–14.5]	11.9 [10.6–12.7]	12.9 [12.9–12.9]	0.005
Albumin (g/dL)	3.9 [3.7–4.1]	4.1 [3.8–4.3]	3.9 [3.8–4.0]	3.9 [3.6–4.0]	3.8 [3.8–3.8]	0.119
Fractional excretion uric acid (%)	6.76 [4.80–8.77]	6.79 [4.97–8.83]	6.27 [4.07–6.84]	7.42 [5.03–9.62]		0.578
Urine protein-to-creatinine ratio (g/gCr)	0.62 [0.09–1.63]	0.13 [0.05–0.83]	0.38 [0.07–1.07]	1.13 [0.34–2.48]	2.77 [2.77–2.77]	0.017
Medication						
Renin-angiotensin system inhibitor (%)	53 (70.7)	12 (57.1)	17 (70.8)	23 (79.3)	1 (100.0)	0.346
Diuretics (%)	16 (21.3)	3 (14.3)	7 (29.2)	6 (20.7)	0 (0.0)	0.62
Anti hyperuricemic agents (%)	38 (50.7)	6 (28.6)	14 (58.3)	17 (58.6)	1 (100.0)	0.095

The median age was 67 years, and 72.0% of the patients were men. 23 (30.7%) of patients had diabetes mellitus and all patients were Japanese. The median eGFR was 35.7 mL/min/1.73 m^2^ and median serum UA level was 6.4 mg/dL. There was no significant difference in the use of antihyperuricemic agents and serum UA levels between CKD stages.

### Study 1: change in serum UA levels by CKD stage

**Figure 2:**
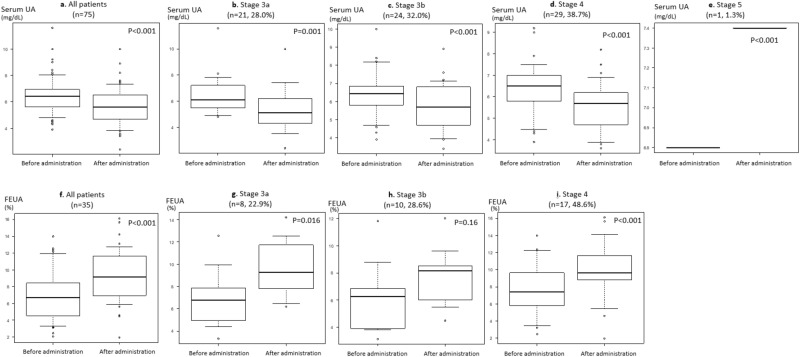
Change in serum UA and FEUA at various CKD stages.

Figure 2a shows a comparison of serum UA levels before and after dapagliflozin administration. A significant decrease in serum UA level (before administration; 6.4 [5.6–7.0] mg/dL vs. after administration; 5.6 [4.7–6.5] mg/dL, *p* < 0.001) was observed before and after dapagliflozin administration. Rate of UA change was − 0.12 [− 0.20 to − 0.04]% by SGLT2i administration.

Figure 2b–e shows the group differences in serum UA changes by CKD stages.

Multiple regression analysis did not reveal a relationship between eGFR, change in eGFR and rate of UA change (Table 2).

**Table 2:** Indicators of rate of change in serum UA level.

	Univariate analysis		Multivariate analysis	
	β	*p* value	β	*p* value
Estimated glomerular filtration rate (ml/min/1.73m^2^)	− 0.172	0.139	− 0.181	0.117
Change of estimated glomerular filtration rate (ml/min/1.73m^2^)	− 0.187	0.109	− 0.187	0.153
Uric acid (mg/dL)	− 0.142	0.224	− 0.142	0.306
Male	− 0.017	0.886	− 0.017	0.957
DM	0.221	0.056	0.222	0.081

### Study 2: change in FEUA by CKD stage

Compared with FEUA before SGLT2i administration, a significant increase in FEUA was observed after dapagliflozin administration (*p* < 0.001) (Fig. 2f).

After dapagliflozin administration, FEUA was changed 6.76 [4.95–7.38]% to 9.28 [8.43–11.73]%, 6.27 [4.07–6.84]% to 8.15 [6.19–8.52]% and 7.42 [5.81–9.62]% to 9.59 [8.80–11.63]% in CKD stage 3a, 3b and 4, respectively (Fig. 2g–i).

Furthermore, multiple regression analysis did not reveal a significant association between eGFR and rate of FEUA change after dapagliflozin administration (Table 3).

**Table 3:** Indicators of rate of change in FEUA.

	Univariate analysis		Multivariate analysis	
	β	*p* value	β	*p* value
Estimated glomerular filtration rate (ml/min/1.73m^2^)	0.252	0.145	5.412	0.199
Uric acid (mg/dL)	− 0.347	0.041	− 0.334	0.059
DM	− 0.106	0.545	0.079	0.671

**Figure 3:**
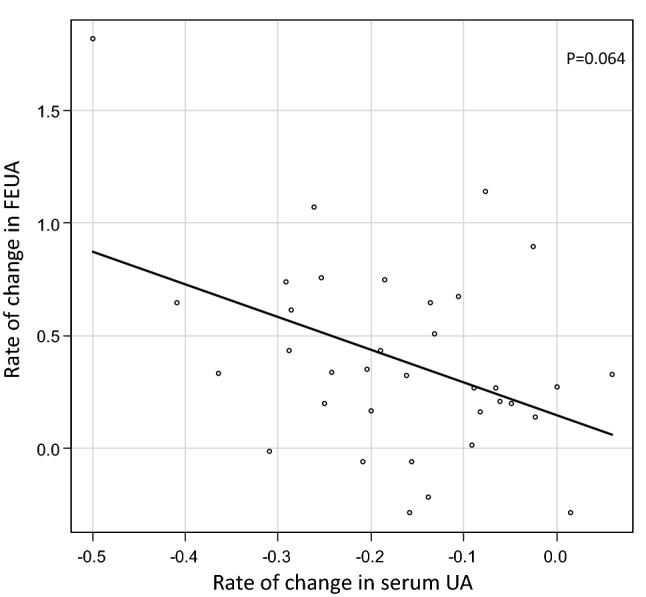
Correlation between change in serum UA and FEUA.

Rate of FEUA change did not correlate with rate of serum UA change (Fig. 3).

## Discussion

Our study showed that there was an increase in FEUA and a decrease in serum UA levels in patients with advanced CKD after dapagliflozin administration compared to the values before administration.

Serum UA levels have been shown to be associated with CKD progression^22,23^. In addition, allopurinol has been demonstrated to lower serum UA levels and slow CKD progression^10,11^. However, there are some reports that allopurinol did not improve renal outcomes^12,13^. Control of serum UA is important for patients with CKD; however, many patients fail to reach the target serum UA level^24^. Post-hoc analysis of CANVAS trial revealed that renal function of patients who received canagliflozin was better than that of patients who received placebo, even in patients with urine albumin-creatinine ratio ˂30 mg/gCr^25^. The renoprotective effect of SGLT2i may not be due to lowering of urinary protein alone, as one of the pleiotropic effects of SGLT2i is reduction of serum UA levels, which may lead to cardiac and renal protection^9^.

Similar to our result, previous studies have shown that SGLT2i increase FEUA and decrease serum UA levels in patients with type 2 DM^14–17^. Ohashi et al. reported that SGLT2i increased FEUA (before administration; 5.98 ± 2.59% vs. after administration; 7.71 ± 3.22%) and reduce serum UA levels (before administration; 6.13 ± 1.36 mg/dL vs. after administration; 5.20 ± 1.11 mg/dL) in patients with type 2 DM whose eGFR was 63.25 ± 24.66 mL/min/1.73 m^2^^17^. Our results in patients with CKD stage 1 and 2 showed that there was a decrease in serum UA levels and an increase in FEUA after dapagliflozin administration same as previous studies (Supplementary Table 1 and Supplementary Fig. 1)^14–17^.

Furthermore, our study revealed that levels of serum UA decreased (before administration: 6.2 [5.5–6.9] mg/dL vs. after administration: 5.4 [4.7–6.1] mg/dL) and FEUA increased (before administration: 6.70 [4.22–8.37]% vs. after administration: 8.77 [6.44–8.37]%) in patients with CKD without DM (Supplementary Table 2 and Supplementary Fig. 2). The impact of SGLT2i on UA may not depend on renal function or DM.

Typically, serum UA is higher in male than in female. Our study showed that SGLT2i reduce serum UA both in male and female patients (Supplementary Fig. 3).

The mechanism underlying SGLT2i’s lowering of serum UA levels has not been clearly elucidated.

URAT1 is already known as uric acid transporter expressed in proximal tubule^26^. Nokikov et al. reported that URAT1 and GLUT9 are required for the increase in FEUA in response to canagliflozin^27^.

Chino et al. reported that increased urinary UA excretion may be attributed to glycosuria caused by SGLT2 inhibitors on GLUT9 isoform 2 or any other transporter(s) at the proximal tubule, and may inhibit uric acid reabsorption mediated by GLUT9 isoform 2 at the collecting duct of the renal tubule^18^.

Although SGLT2i increased FEUA and decreased serum UA in our study, change in FEUA and change in serum UA was not associated with eGFR. Furthermore, we could not find a significant association between change in FEUA and change in serum UA.

Mechanisms other than urinary excretion such as intestinal excretion of UA may be involved in lowering effects of SGLT2i on serum UA levels in patients with advanced CKD.

ABCG2 is a high-capacity urate secretion transporter^28^. In animal studies, intestinal urate transport by ABCG2 compensated for abnormal urate handling in the setting of declining renal function^19^. Lu et al. reported that empagliflozin promoted ABCG2 expression in the kidneys and ileum of diabetic mice and attenuated hyperuricemia^29^. Although these reports suggest that SGLT2i increase urinary UA excretion and promote intestinal extrusion of uric acid through UA transporters, no obvious evidence has yet been found.

Further studies are needed to demonstrate the underlying mechanism of SGLT2i’s reduction of serum UA levels.

## Limitations

The present study had some limitations. First, this was a single-center retrospective cohort study with a relatively small sample size. Second, we did not investigate the dietary characteristics of each patient. Third, because we did not assess difficult endpoints such as ESRD and CKD progression, we did not show whether reduction of UA by SGLT2i may improve renal outcomes.

## Conclusion

Our results suggest that dapagliflozin increases FEUA and decreases serum UA levels in patients with advanced CKD.

However, the mechanism of the effect of dapagliflozin on lowering serum UA level has not been fully elucidated, and it is not clear whether this effect can improve renal outcomes. Therefore, further studies are needed to identify the mechanism and impact of SGLT2i on renal outcomes by lowering of serum UA levels.

## Supplementary Information

Supplementary Information.

## Data Availability

All data are available in the main text or supplementary materials.
