# Supplementary material for: The effect of dapagliflozin on uric acid excretion and serum uric acid level in advanced CKD
Source: Sci Rep. 2023 Mar 24;13:4849. doi: 10.1038/s41598-023-32072-y (PMC10039024; doi:10.1038/s41598-023-32072-y)
Supplement: Supplementary file 1 — Supplementary Information. [file 41598_2023_32072_MOESM1_ESM.pdf]

The effect of dapagliflozin on uric acid excretion and serum uric acid level in advanced CKD

Yukimasa Iwata, MD; Shoki Notsu, MD; Yushi Kawamura, MD; Waka Mitani, MD; Shinjiro Tamai, MD; Madoka Morimoto, MD; *Masafumi Yamato, MD, PhD

Supplementary Table1. Patient characteristics and laboratory data at dapagliflozin 10mg administration in patients with CKD stage 1 and 2

	All patients n=12	CKD stage 1 n=2	CKD stage 2 n=10
Male (%)	6 (50.0)	2 (100.0)	4 (40.0)
Age	48 [39-68]	73 [71-76]	46 [38-52]
**Comorbid condition**			
Heart failure (%)	0 (0.0)	0 (0.0)	0 (0.0)
Diabetic mellitus (%)	1 (8.3)	0 (0.0)	1 (10.0)
**Clinical and laboratory variables**			
Systolic blood pressure (mmHg)	134 [125-137]	135 [135-135]	132 [125-139]
Diastolic blood pressure (mmHg)	75 [70-78]	70 [70-70]	75 [70-79]
Pulse rate (bpm)	75 [71-81]	75 [68-81]	75 [68-81]
Creatinine (mg/dL)	0.76 [0.66-0.83]	0.60 [0.57-0.62]	0.78 [0.74-0.86]
Estimated glomerular filtration rate (ml/min/1.73㎡)	73.9 [66.1-78.4]	100.8 [95.7-106.0]	72.0 [65.6-74.7]
Blood urea nitrogen (mg/dL)	13 [12-15]	19 [17-21]	12 [12-14]
Uric acid (mg/dL)	5.5 [4.0-6.7]	6.1 [5.2-7.1]	5.5 [3.5-6.5]
Hemoglobin (g/dL)	13.4 [12.9-14.9]	15.3 [14.1-16.4]	13.4 [12.9-14.3]
Albumin (g/dL)	3.7 [3.5-4.1]	3.4 [3.4-3. 5]	3.9 [3.6-4.2]
Fractional excretion uric acid (%)	3.97 [2.68-7.02]	2.08 [2.08-2.08]	5.49 [3.64-7.35]
Urine protein-to-creatinine ratio (g/gCr)	0.75 [0.26-2.31]	4.98 [4.01-5.95]	0.30 [0.22-0.99]
**Medication**			
Renin-angiotensin system inhibitor (%)	10 (83.3)	2 (100.0)	8 (80.0)
Diuretics (%)	0 (0.0)	0 (0.0)	0 (0.0)
Anti hyperuricemic agents (%)	1 (8.3)	0 (0.0)	1 (10.0)

Data given as median (IQR). Abbreviation: CKD-chronic kidney disease

Supplementary Figure 1.

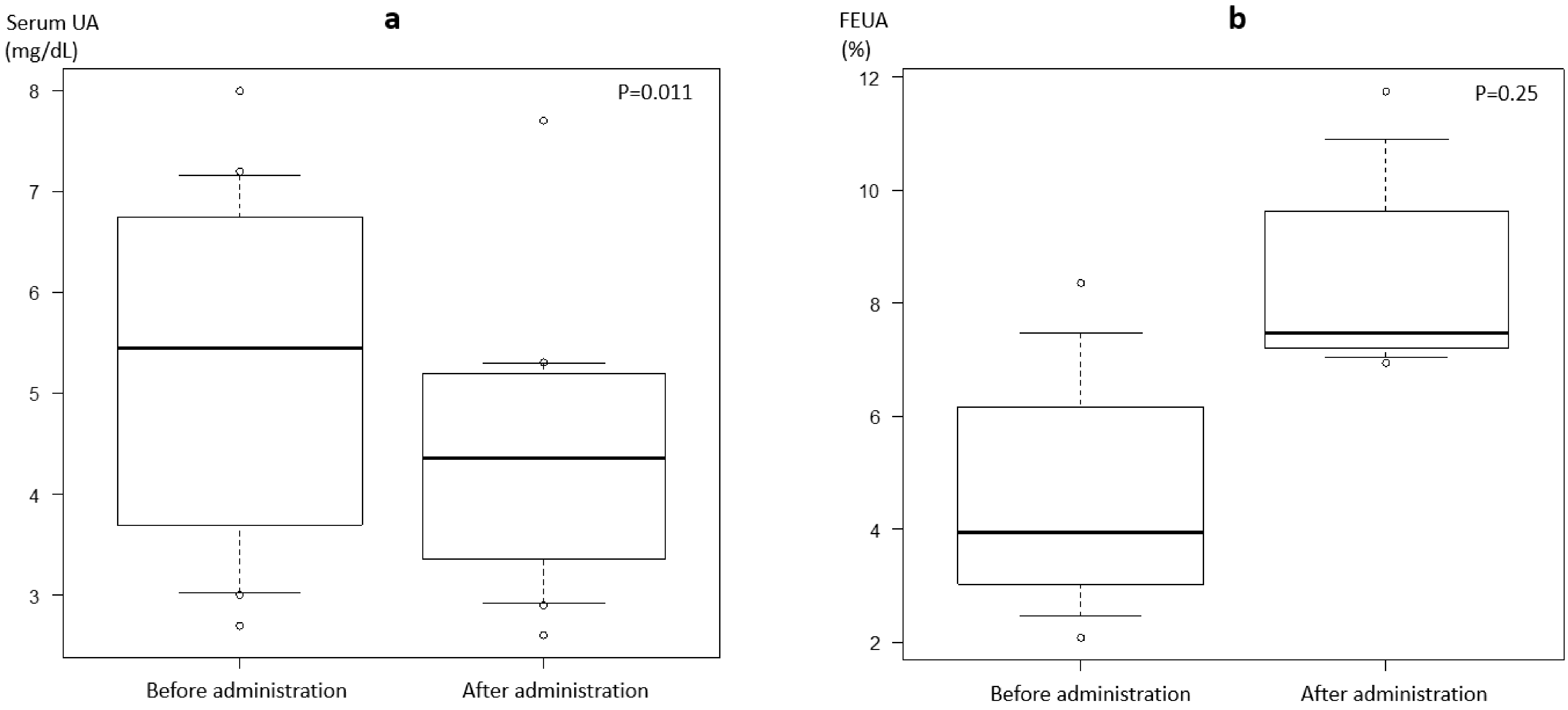

Change in **a** serum UA and **b** FEUA in patients with CKD stage 1 and 2

Supplementary Table2. Patient characteristics and laboratory data at dapagliflozin 10mg administration with or without DM

	nonDM n=52	DM n=23	p value
Male (%)	34 (65.4)	20 (87.0)	0.092
Age	66 [57-77]	68 [56-75]	0.713
**Comorbid condition**			
Heart failure (%)	3 (5.8)	0 (0.0)	0.548
Diabetic mellitus (%)	52 (100.0)	0 (0.0)	<0.001
**Clinical and laboratory variables**			
Systolic blood pressure (mmHg)	131 [120-140]	135 [125-142]	0.395
Diastolic blood pressure (mmHg)	70 [66-80]	77 [70-84]	0.295
Pulse rate (bpm)	75 [70-83]	89 [70-94]	0.104
Creatinine (mg/dL)	1.42 [1.19-1.94]	1.60 [1.30-1.96]	0.373
Estimated glomerular filtration rate (ml/min/1.73㎡)	35.4 [26.2-46.9]	36.9 [26.6-44.4]	0.899
Blood urea nitrogen (mg/dL)	23 [17-29]	25 [20-33]	0.373
Uric acid (mg/dL)	6.2 [5.5-6.9]	6.5 [6.1-7.0]	0.37
Hemoglobin (g/dL)	12.8 [11.8-14.0]	12.0 [10.9-13.0]	0.198
Albumin (g/dL)	4.0 [3.7-4.1]	3.8 [3.7-4.0]	0.217
Fractional excretion uric acid (%)	6.70 [4.22-8.37]	7.57 [5.81-11.82]	0.171
Urine protein-to-creatinine ratio (g/gCr)	0.59 [0.08-1.52]	0.71 [0.11-2.56]	0.527
**Medication**			
Renin-angiotensin system inhibitor (%)	34 (65.4)	19 (82.6)	0.173
Diuretics (%)	7 (13.5)	9 (39.1)	0.029
Anti hyperuricemic agents (%)	27 (51.9)	11 (47.8)	0.805

Data given as median (IQR). Abbreviation: CKD-chronic kidney disease

Supplementary Figure 2.

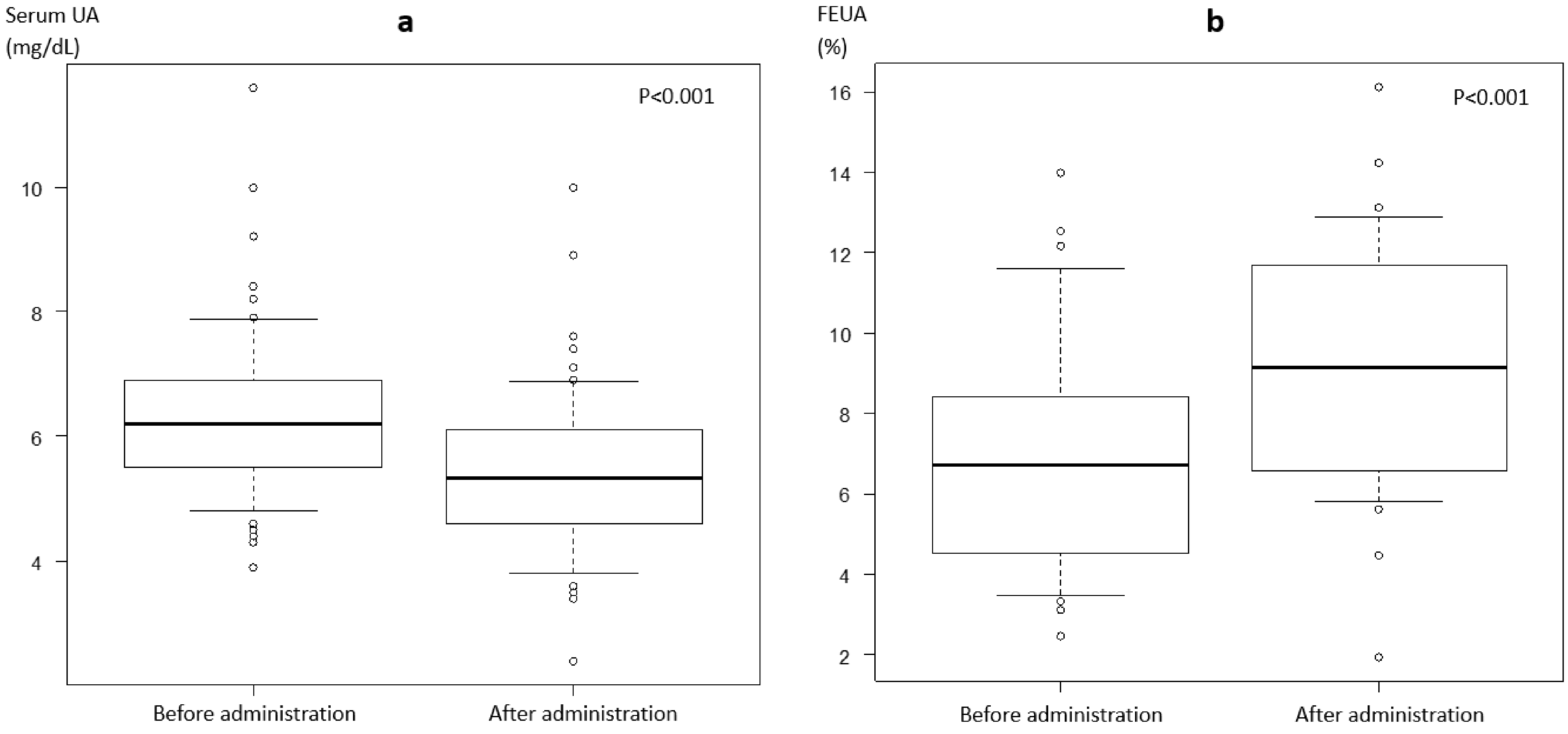

Change in **a** serum UA and **b** FEUA in patients without DM

Supplementary Figure 3.

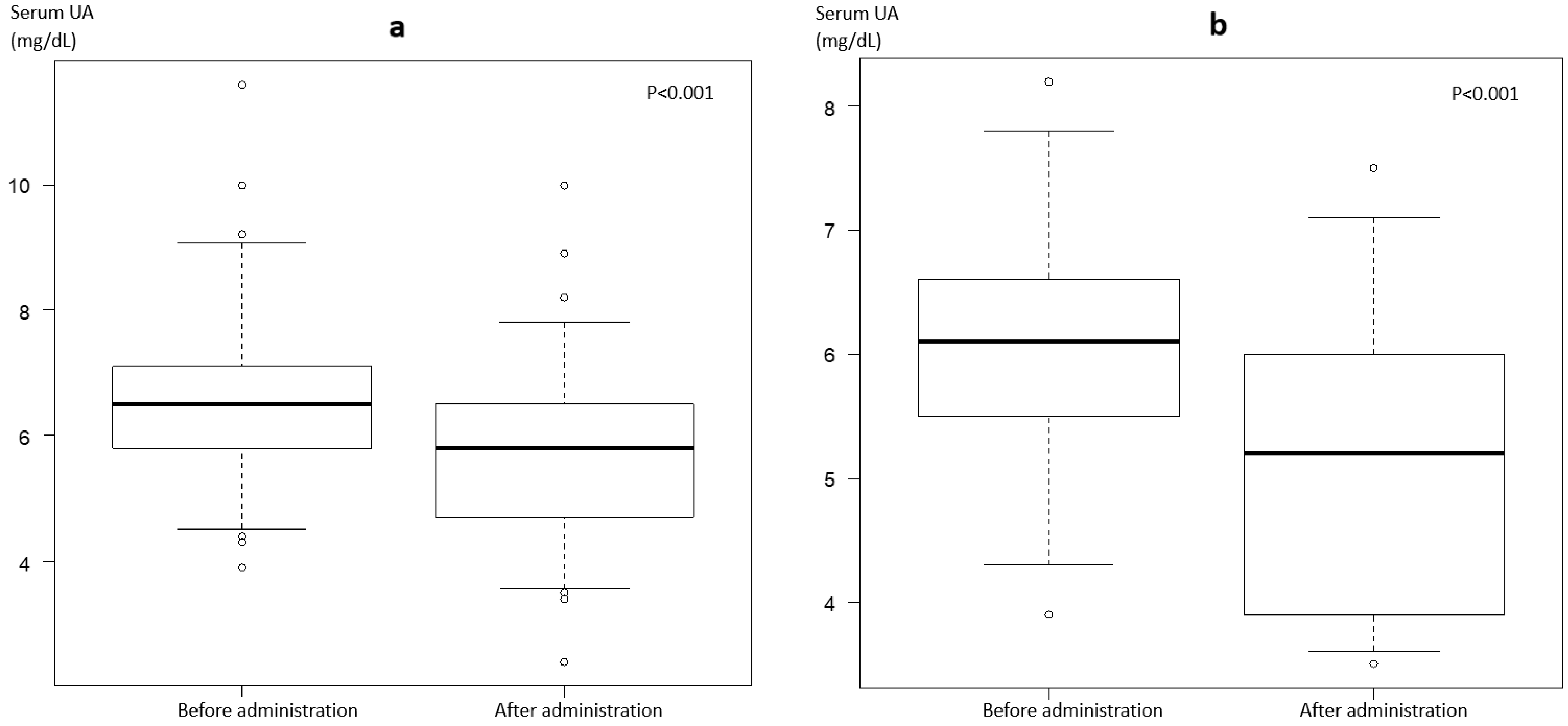

Change in serum UA in **a** male and **b** female
